# Late-stage anterior cruciate ligament reconstruction rehabilitation in the United Kingdom: an online survey of National Health Service physiotherapists

**DOI:** 10.1186/s13102-025-01438-2

**Published:** 2025-11-22

**Authors:** Sarah Jones, Gareth Stephens, Michael Dawes, Michael Mansfield, Matthew Willett

**Affiliations:** 1 NHS St George’s University Hospitals Foundation Trust, London, England SW17 0QT UK; 2 NHS University Hospitals of Liverpool, Liverpool, England L7 8YE UK; 3 Research and Development, The Royal Orthopaedic Hospital NHS Foundation Trust, Northfield, Birmingham, B31 2AP UK; 4 Centre For Musculoskeletal Medicine, Royal Orthopaedic Hospital NHS Foundation Trust, Bristol Road South, Northfield, Birmingham, B31 2AP UK; 5 School of Sport, Exercise and Rehabilitation Sciences, College of Life and Environmental Sciences, University of Birmingham, Edgbaston, Birmingham, B15 2TT UK; 6 Centre of Precision Rehabilitation for Spinal Pain, University of Birmingham, Edgbaston, Birmingham, B15 2TT UK

**Keywords:** Anterior cruciate ligament reconstruction, Return to sports, Physiotherapy, Late-stage rehabilitation, National health service

## Abstract

**Background:**

Late-stage Anterior Cruciate Ligament reconstruction (ACL-R) rehabilitation aims to help patients to return to sports (RTS) and their previous activity levels and minimise reinjury rates. In the United Kingdom, National Health Service (NHS) physiotherapists lead post-ACL-R rehabilitation programs. This survey aimed evaluate NHS physiotherapists’ perspectives of the resources available, and their confidence to deliver, late-stage ACL-R rehabilitation. The secondary aim was to identify the RTS tests conducted by NHS physiotherapists on ACL-R patients.

**Methods:**

An online cross-sectional survey of NHS physiotherapists was conducted. Proportions and percentages summarised respondents’ perspectives of the resources (equipment, space, and time) available, and their confidence, to deliver late-stage ACL-R rehabilitation components (strength testing, Neuromuscular control, movement quality analysis, plyometrics, sports specific drills, and psychological readiness evaluation) and RTS testing. Spearman signed rank test evaluated the strength and direction of any correlation between physiotherapists’ perspectives of available resources, clinical experience, and their confidence to perform late-stage rehabilitation. The RTS tests used across each component of late-stage ACL-R rehabilitation were recorded.

**Results:**

102 physiotherapists completed the online survey between June-July 2022. Approximately half of respondents (53%) believed most ACL-R patients (>60%) RTS. Approximately half to three quarters of respondents agreed that there were adequate resources available to perform strength (range of 49-60% respondents agreed), neuromuscular control (63-72%), movement quality analysis (53-56%) and plyometric (48-52%) components of late-stage ACL-R rehabilitation. Less than half of respondents agreed there were sufficient resources to perform sports specific drills (18%-38%), psychological readiness evaluation (20-46%), and RTS testing (26%-33%). Most respondents were confident to deliver the exercise-based components of ACL-R late-stage rehabilitation (e.g. strength testing; range 68-92%) and decide when patients were ready to RTS (75%). Conversely, most respondents were not confident to perform psychological readiness evaluation, which was moderately positively correlated with their perspectives of resources. Respondents used a wide variety of non-evidence based RTS tests across all components of late-stage ACL-R rehabilitation.

**Conclusion:**

Targeted training in evidence based late-stage rehabilitation ACL-R protocols and improved access to appropriate resources maybe required to enable NHS physiotherapists to deliver optimal late-stage ACL-R rehabilitation.

**Trial registration:**

N/A

**Supplementary Information:**

The online version contains supplementary material available at 10.1186/s13102-025-01438-2.

## Introduction

Over 20,000 people who play sports in the United Kingdom (UK) suffer an anterior cruciate ligament (ACL) injury each year [1]. After ACL injury, 75% of UK patients chose to undergo anterior cruciate ligament surgical reconstruction (ACL-R), with the National Health Service (NHS) funding approximately 80% of such procedures at a cost of £60 million per annum [2].

Contemporary guidelines [3, 4] recommend that a multidisciplinary team (e.g. patient, surgeon, physiotherapist, coaches) collaboratively decide when to progress patients through the early, mid, and late-stage phases of ACL-R rehabilitation. However, due to limited resources, NHS ACL-R rehabilitation is primarily delivered by physiotherapists. The incorporation of comprehensive late-stage ACL-R rehabilitation, including return to sport (RTS) testing, is fundamental to successful return to pre-injury levels of activity [5]. While ACL-R rehabilitation phase progression should be informed by patients meeting pre-defined criteria rather than timeframes [6], in physiotherapy practice, late-stage rehabilitation generally occurs approximately six months post ACL-R [7–10]. Late-stage ACL-R rehabilitation incorporates several components that can be characterised as either *exercise-based* or *psychological-based* [11–15]. Exercise-based components of late-stage ACL-R rehabilitation include strength training, neuromuscular training, movement quality analysis, plyometric exercises, sports specific drills, and on-field rehabilitation; Psychological components incorporate psychological readiness evaluation, which are frequently captured by patient reported outcome measures (PROMs).

Most published ACL-R rehabilitation protocols are based on research conducted on elite sporting populations [16–18]. However, NHS departments may have less resources available (e.g. time, space, and equipment) to conduct late-stage ACL-R rehabilitation compared to elite-sport settings [19] which may have consequences for patients, physiotherapists, and the wider healthcare service. For example, limited RTS testing opportunities may partially explain the decreased physiotherapist confidence found when delivering late-stage ACL-R rehabilitation [20] or the lower-proportions of non-elite athletes who RTS [21–23] or their presurgical activity levels [24, 25] compared with elite athletes. Although empirical evidence is lacking [26], ACL-R patients are encouraged to achieve pre-defined functional and psychological criteria prior to RTS to reduce the likelihood of graft failure. Revision ACL-R surgery is more complex than the primary operation and incurs a cost of approximately £7000 per procedure [27, 28]. Furthermore, post-revision patients require further prolonged periods of rehabilitation, absences from work and recreational/sporting activities, and may experience low mood [29].

Despite the volume of late-stage ACL-R rehabilitation conducted within the NHS, NHS physiotherapists’ perspectives on available resources and their confidence to deliver care remain unclear. This data may highlight gaps in service provision and modifiable factors to target when developing interventions that aim to promote RTS and minimise re-rupture rates in ACL-R patients. Therefore, the primary aims of this study were to evaluate NHS physiotherapists’ perspectives of the available resources, and their confidence to deliver, individual components of late-stage rehabilitation and RTS testing in ACL-R patients. The secondary aim was to identify the RTS testing conducted by NHS physiotherapists on ACL-R patients.

**Objectives**:

1. To evaluate NHS physiotherapists’ perspectives of the resources (equipment, space, and time) available, and their confidence to deliver, components of late-stage rehabilitation and RTS testing in ACL-R patients.
2. To identify the components of late-stage rehabilitation and PROMs that NHS physiotherapists utilise to inform their RTS decision making in ACL-R patients.
3. To evaluate the correlation between NHS physiotherapists’ experience (banding and years of post-graduate experience) and their perspectives of resources available with their confidence to deliver individual components of late-stage rehabilitation to ACL-R patients.

## Methods

### Survey development and content

This cross-sectional, online survey was conducted and reported in accordance with the Consensus-Based Checklist for Reporting of Survey Studies guidelines [30] (additional file 1). The survey included 29 questions (additional file 2) with content informed by the study groups clinical experience and contemporary ACL evidence [1, 12, 31–33].

The survey was split into the following sections:

- Section 1 (questions 1–5) included a participant information sheet (PIS) and consent form. Participants were encouraged to download and read the PIS and email any questions to the study team prior to giving informed voluntary consent.
- Section 2 (questions 6–14) obtained physiotherapists’ demographic (e.g. geographic location) and occupational data (e.g. years qualified) and their perspectives on how often and long ACL-R patients require to progress to late-stage rehabilitation and RTS testing.
- Section 3 (questions 14–19; 28–29) evaluated physiotherapists’ perspectives of NHS facilities available (equipment, space, and time), and their confidence to complete late-stage rehabilitation and RTS testing (Objective 1).
- Section 4 (questions 20–27) identified the performance-based tests and PROMs that NHS physiotherapists used to inform RTS decision making (Objective 2).

To aid data interpretation and comparison with other studies, where relevant, questions were subcategorised into core components of late-stage rehabilitation and RTS testing including: strength tests, neuromuscular testing, movement quality analysis, plyometric exercises, sports specific drills, psychological readiness evaluation [1, 12, 31–33]. Component definitions were informed by the evidence and the research teams clinical experience (additional file 4). Strategies related to on-field assessment/rehabilitation were not sought as these are not routinely performed in NHS physiotherapy departments. Survey questions were piloted by a convenience sample of physiotherapists in two NHS physiotherapy departments in separate geographical locations: one in London and one in North West England. Questions were updated prior to the survey’s launch based on the pilot samples feedback.

### Dissemination

The study was approved by the School of Sport, Exercise, and Rehabilitation Sciences Ethics Committee at the University of Birmingham, UK (ref no. MCR2122_24). Purposive sampling targeted NHS physiotherapists who treated ≥ 1 patient post ACL-R per year. A single stage sample was utilised to maximise external validity and response diversity. No power calculation was completed prior to launch.

Physiotherapists were recruited via an online invitation which outlined the survey’s rationale, aims, the study teams contact details, and a survey link. The survey was promoted via social media (Twitter) and professional networks including the Musculoskeletal Association of Chartered Physiotherapists and the Musculoskeletal Network of the Interactive Board of Chartered Society of Physiotherapists. No training was required for physiotherapists to complete the survey.

### Data analysis

The survey utilised a combination of multiple choice, free text, and Likert scales to capture responses (additional file 3). Anonymised data was exported from Microsoft Forms into Microsoft Excel and the Statistical Package for the Social Sciences Version 25 for analysis and securely stored on the Research Electronic Data Capture platform.

#### Descriptive analysis

Descriptive data were reported as proportions (number who responded positively to questions/total number who responded to question) and percentages. Different Likert scales were used to capture respondents’ perspectives of available resources, and confidence to deliver late-stage rehabilitation (Objective 1) and their use of specific component RTS tests (Objective 2). In line with other contemporary physiotherapy surveys [34, 35], responses from Likert scales were dichotomised to simplify interpretation of descriptive data. Details on how each Likert scales were utilised and interpreted has been summarised in Table 1.

**Table 1:** Likert scales utilised and interpretation in descriptive analysis

Research objective	Likert scale utilised	Dichotomisation of responses for descriptive analysis
Perspectives of the adequacy of resources available to complete components of late-stage rehabilitation	**Five-point agreement scale**: ‘Strongly agree’, ‘Agree’, ‘Neutral’, ‘Disagree’, ‘Strongly Disagree’.	**Strongly agree/Agree**: Respondent **‘agreed’** that resource was adequately available to conduct component late-stage rehabilitation **Neutral/Disagree/Strongly Disagree**: Respondents **‘did not agree’** that resource was adequately available to conduct component of late-stage rehabilitation
Confidence to deliver components of late-stage rehabilitation	**Five-point confidence scale**: ‘Very Confident’, ‘Confident’, ‘Neutral’, ‘Unconfident’, ‘Very Unconfident’).	**Very confident/confident**: Respondent **‘felt confident’** to perform component of late-stage rehabilitation **Neutral**,** Unconfident**,** Very Unconfident**: Respondents **‘did not feel confident’** to perform component of late-stage rehabilitation.
Performance-based tests and PROMs used during RTS testing	**Three-point frequency scale**: ‘yes’, ‘sometimes’, ‘no’.	**Yes**: Respondent **‘used’** specified Component/PROM **Sometimes/No**: Respondents **‘did not use’** specified component/PROM

*PROM* Patient Reported Outcome Measure, *RTS* Return to Sport

#### Inferential analysis

To evaluate the statistical strength and direction of any relationship that existed between respondents’ experience and their perspectives of the available resources and confidence to deliver late-stage rehabilitation, Spearman’s rho was calculated for ordinal data (Objective 3). Statistical significance was set at *p* < 0.05 and correlation strength was interpreted as follows: 0: No correlation; +/- 0.1–0.3: Weak correlation: +/- 0.4–0.6: Moderate correlation; +/−0.7−0.9: Strong correlation; +/- 1: Perfect correlation.

## Results

During social media promotion, the tweet was circulated (i.e. re-tweeted) 50 times, the survey link was opened 58 times, and the tweet was engaged with (interaction by clicking anywhere on the tweet, likes, follows, replies) 421 times. Although all questions received responses, not all respondents answered each question. Therefore, the ratio (defined as number of respondents who provided a positive response to the question/number of respondents who answered the question) and the percentage is reported to support the descriptive analysis.

### Study uptake and respondents’ demographic features

**Fig. 1:**
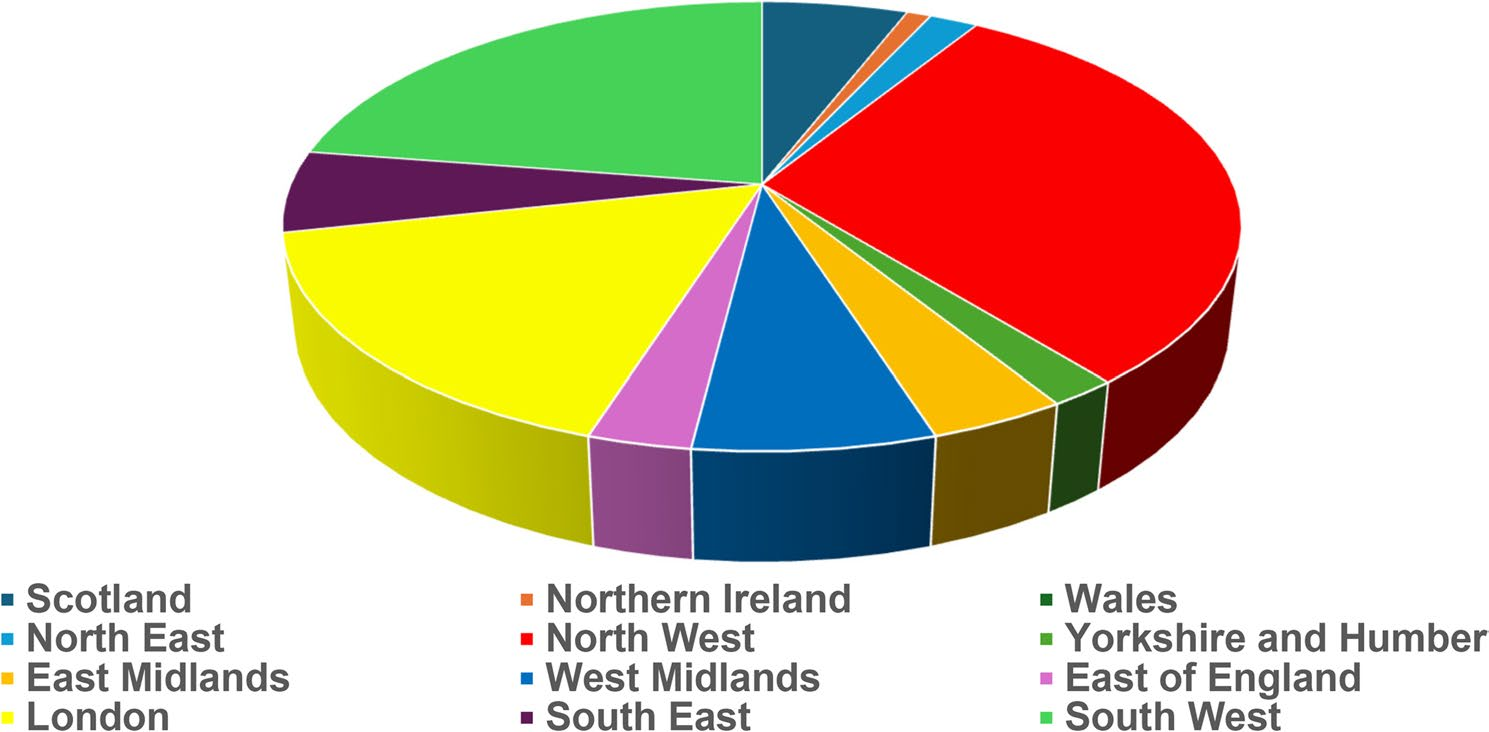
National health service band and geographical location of respondents

Between June-July 2022, 102 registered physiotherapists (respondents) across NHS bands 5–8 undertook the survey (Fig. 1A). Most respondents worked at either band 6 (*n* = 36/102; 35%) or 7 (*n* = 39/102; 38%) levels while band 5 was underrepresented (*n* = 3/102; 3%). The most common category of respondent post-graduate experience was 3–6 years (*n* = 28/102; 27%) with 0–3 years’ experience (*n* = 9/102; 9%) the fewest. There was a relatively even spread across other experience timeframes. Almost all respondents worked in England (*n* = 96/102; 94%) with the highest number working in the North West (*n* = 31/102; 30%), the South West (*n* = 23/102; 23%), and London (*n* = 17/102;17%) respectively. The remaining respondents (*n* = 6/102; 6%) worked in Scotland (Fig. 1B). Most respondents (*n* = 87/102; 85%) spent less than 25% of their clinical time treating ACL-R patients. Over half of respondents (*n* = 57/102; 56%) had a special interest in ACL-R rehabilitation and most were educated to either a Bachelors (*n* = 55/102; 54%) or Masters (*n* = 39/102; 38%) level. The demographic details of respondents are presented in Table 2.

**Table 2:** Respondents professional demographic data

**Employment**				
**NHS Band**	***N (%)***		**Years of post graduate experience**	***N (%)***
5	3 (3%)		Less than 3 years	9 (9%)
6	36 (35%)		3–6 years	28 (27%)
7	39 (38%)		7–10 years	19 (19%)
8	24 (24%)		11–15 years	17 (17%)
			16–20 years	13 (13%)
			Over 20 years	16 (16%)
**Education and professional interest**				
**Highest Educational Award**		**N (%)**	**Special interest in ACL rehabilitation**	**N (%)**
BSc/Graduate Diploma Physiotherapy		55 (54%)	Yes	57 (56%)
BA		1 (1%)	No	45 (44%)
MSc (pre-registration)		15 (15%)		
Post Graduate Certificate		5 (5%)		
Post Graduate Diploma		6 (6%)		
MSc (post-registration)		23 (23%)		
MPhil		1 (1%)		
PhD		1 (1%)		
Other		1 (1%)		

*NHS* National Health Service, *N* Number, 5% percentage of respondents, *BSC* Bachelor of Science, *BA* Bachelor of Arts, *MPhil* Master of Philosophy, *PhD* Doctor of Philosophy, *ACL* Anterior Cruciate Ligament

### Respondents’ perspectives of the NHS resources available and confidence to conduct late-stage rehabilitation and return to sport testing (Objective 1)

**Fig. 2:**
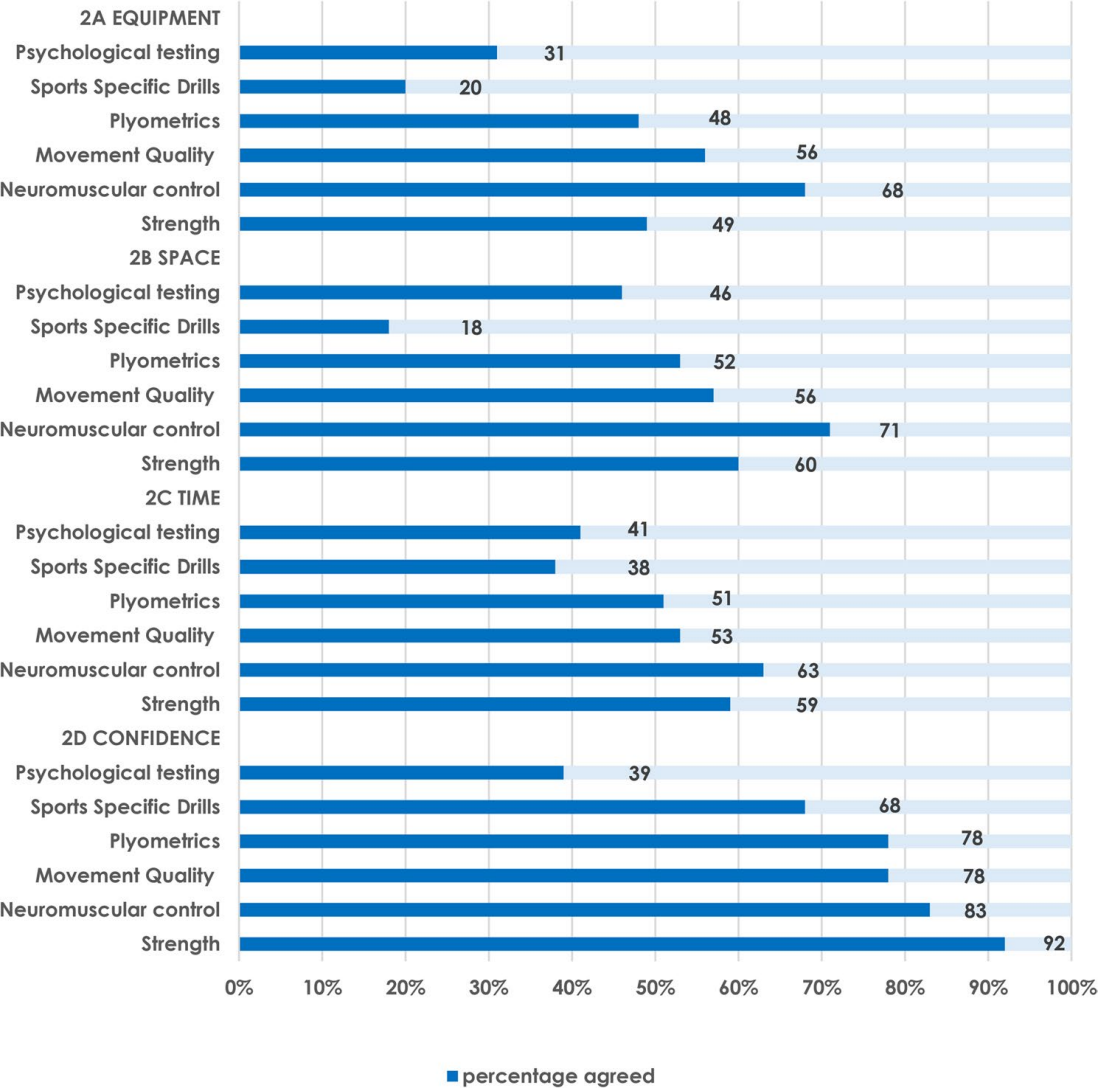
Respondents perspectives of resources available and confidence to deliver components of late-stage rehabilitation

Respondents’ perspectives of the time, space, and equipment available, and their confidence, to complete each component of late-stage ACL-R rehabilitation and RTS testing within the NHS is reported below and summarised in Fig. 2 (A-D) and additional files 2 and 3 (questions 14–18; 22) respectively.

#### Return to sport testing

Approximately one quarter to one third of respondents agreed they had the necessary equipment (*n* = 26/102; 26%), space (*n* = 28/102/27%), and time (*n* = 34/102; 33%) to complete RTS testing.

#### Equipment

Most responders agreed they had appropriate equipment to conduct late-stage neuromuscular control (*n* = 69/102; 68%) and movement quality analysis (*n* = 57/102; 56%). Approximately one half of respondents agreed they could complete strength (*n* = 50/102; 49%) and plyometric (*n* = 49/102; 48%) exercises. Less than one third of respondents agreed they could conduct psychological readiness evaluation (*n* = 20/102; 20%) or sports specific drills (*n* = 32/102; 31%).

#### Space

Most respondents had access to a gym (*n* = 86/102; 85%) to support late-stage rehabilitation, with approximately one half of these (*n* = 53/102; 52%) also offering an ACL-R rehabilitation class. Most respondents felt they had adequate space to conduct late-stage neuromuscular control, (*n* = 72/102; 71%), strength (*n* = 61/102; 60%), movement quality analysis (*n* = 57/102; 56%), and plyometric (*n* = 53/102, 52%) rehabilitation. Less than half of the respondents felt they had adequate space to complete psychological readiness evaluation (*n* = 47/102; 46%) and few felt they could complete sports specific drills (*n* = 18/102; 18%).

#### Time

Over half of respondents agreed they had enough time to perform late-stage neuromuscular control (*n* = 64/102; 63%), strength (*n* = 60/102; 59%), movement quality (*n* = 54/102; 53%), and plyometric (*n* = 52/102; 51%) rehabilitation respectively. Less than half of respondents agreed they had adequate time to complete psychological readiness evaluation (*n* = 42/102; 41%) and sports specific drills (*n* = 39/102; 38%).

#### Confidence

Approximately half of the respondents (*n* = 54/102; 53%) self-reported that > 60% of ACL-R patients progress to late-stage rehabilitation and RTS. Most respondents felt that ACL-R late-stage rehabilitation commenced a minimum of 6 months post-surgery (*n* = 88/102; 86%) and took at least 10 months to RTS (*n* = 85/102; 83%). Overall, three quarters of respondents (*n* = 77/102; 75%) were confident to decide when ACL-R patients were ready to RTS. Most physiotherapists felt confident to conduct late-stage strength (*n* = 94/102; 92%), neuromuscular control (*n* = 85/102; 83%), movement quality analysis (*n* = 80/102; 78%), plyometrics (*n* = 80/102; 78%) and sports specific drills (*n* = 69/102; 68%) respectively. However, less than half of respondents were confident to deliver psychological readiness evaluation (*n* = 40/102; 39%). Most respondents agreed that using PROMs (*n* = 72/102; 70%) or psychological readiness evaluation (*n* = 57/102; 56%) improved their confidence when making RTS decisions.

Less than one half of respondents agreed that utilising a rehabilitation protocol improved their confidence to complete late-stage strength (*n* = 48/102; 47%), plyometrics (*n* = 44/102; 43%), neuromuscular control (*n* = 39/102; 38%), movement quality (*n* = 37/102; 36%), sports specific drills (31/102; 30%), or psychological readiness evaluation (*n* = 25/102; 25%) respectively.

### Component methods used to inform return to sport decision making (Objective 2)

**Fig. 3:**
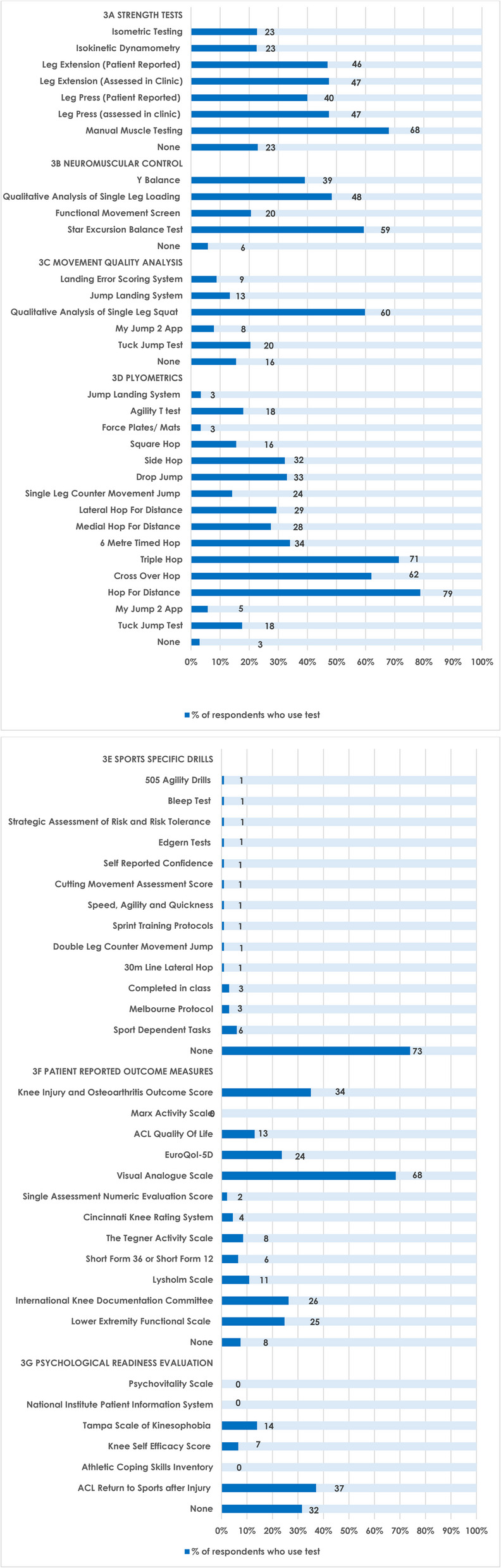
Respondents methods of return to sport tests

The individual component methods used by respondents to inform RTS decision making are summarised below (Fig. 3A-G and Additional files 2 and 3; Questions 20–21;23–27).

#### Strength

Most respondents (*n* = 68/100; 68%) used manual muscle testing (MMT) to evaluate strength during RTS testing. Less than one half of respondents used a version of the leg-press (clinically assessed: *n* = 46/97; 47%; patient reported: *n* = 38/95; 40%) or leg extension (clinically assessed *n* = 46/96; 48%; patient reported: *n* = 44/96; 46%) exercises respectively. Less than one quarter of respondents used isokinetic dynamometry (IKD) (*n* = 21/93; 23%) or isometric testing/hand-held dynamometry (*n* = 22/97; 23%).

#### Neuromuscular testing

Most respondents used the Star Excursion Balance Test (*n* = 57/96; 59%) in RTS testing. Less than half respondents used the Qualitative analysis of single leg loading (*n* = 45/93; 48%) or the Y-balance test (*n* = 36/92; 39%) and approximately one-fifth used functional movement screening (*n* = 20/97; 21%).

#### Movement quality analysis

Most respondents used the Qualitative Analysis of Single leg Squat (*n* = 55/92; 60%) to assess movement quality during RTS testing. Other tests, including the Tuck Jump Test (*n* = 19/93; 20%), My Jump 2 app (*n* = 7/89; 8%), Jump landing system (*n* = 12/90; 13%) and the Landing Error Scoring System (*n* = 8/91; 9%) were infrequently used by respondents.

#### Plyometrics

Most respondents used the Hop for Distance (*n* = 78/99: 79%), Triple Hop (*n* = 70/98; 71%) and Cross Over Hop (*n* = 62/100; 62%) during RTS testing. Approximately one-quarter to one-third of respondents used the Drop Jump (*n* = 30/91; 33%), Side Hop (*n* = 30/93; 32%), Six Metre Hop (*n* = 32/94; 34%), Medial Hop (*n* = 25/91; 27%) or Lateral hop (*n* = 27/92; 29%) tests respectively. Respondents rarely used the Jump Landing System (*n* = 3/89; 3%), Force Plates/Mats (*n* = 3/90; 3%), My Jump 2 App (*n* = 5/87; 6%), Single Leg Counter Movement Jump (*n* = 13/92; 14%), Tuck Jump Test (*n* = 16/91; 18%), Square Hop (*n* = 14/90; 16%), or Agility T tests (*n* = 16/89; 18%) respectively.

#### Sports specific drills

Almost three-quarters of respondents did not use any SSDs (*n* = 74/102; 73%) during RTS testing. There was great variability over SSDs used, with the most common including: Non-specific drills (6/102; 6%), the Melbourne RTS protocol (3/102; 3%), and testing within exercise classes (3/103; 3%). Several unique sports specific drills were suggested by respondents (Additional files 2–3 question 27).

Psychological readiness evaluation Almost one-third of respondents (*n* = 24/76; 32%) did not use a psychological readiness scale to assess RTS. Over one-third of respondents used the Anterior Cruciate Ligament Return to Sports after Injury (ACL-RSI) (*n* = 36/97; 37%) scale within RTS testing. Other tests were rarely/not used by respondents.

#### Patient reported outcome measures

Most respondents used the visual analogue scale (VAS) (*n* = 67/98; 68%). Several PROMs were used by approximately one-quarter to one-third of respondents, including: the Knee Injury and Osteoarthritis Outcome Score (*n* = 34/97; 35%), International Knee Documentation Committee (IKDC; *n* = 25/95; 26%), Lower Extremity Functional Scale (*n* = 23/97; 25%), and the EuroQol-5D (*n* = 22/93; 24%). Other PROMs were rarely used by respondents.

### Correlation between respondents’ confidence to deliver late-stage rehabilitation and their perspectives of resource availability and professional experience (Objective 3)

Moderate-positive correlations were observed between respondents’ confidence to perform Psychological Readiness Evaluation and their perspectives of the time (r_s_=0.360, *P* < 0.010), space (r_s_=0.426, *P* < 0.001), and equipment (r_s_=0.446, *P* < 0.001) available. Weak positive correlations were found between respondents’ confidence to perform neuromuscular control and time available (r_s_=0.205, *P* = 0.039), and confidence to perform movement quality analysis and time (r_s_=0.294, *P* = 0.003) and space (r_s_=0.240, *P* = 0.015) available respectively.

NHS banding was moderately-positively correlated with confidence to perform neuromuscular control (r_s_= 0.408, *P* < 0.01) and movement quality analysis (r_s_= 0.329, *P* < 0.01) and weakly-positively correlated with strength (r_s_= 0.214, *P* = 0.031), plyometrics (r_s_= 0.238, *P* = 0.016) and psychological readiness evaluation (r_s_= 0.237, *P* = 0.017). A weak positive correlation was found between years of post-graduate experience and confidence to perform of neuromuscular control (r_s_= 0.262, *P* = 0.008) and movement quality analysis (r_s_= 0.256, *P* = 0.009). The results of the correlation analysis between respondents’ confidence to deliver late-stage rehabilitation and their perspectives of resources available and professional experience is presented in Table 3.

**Table 3:** Correlation of respondents’ confidence to deliver late-stage rehabilitation with perspectives of available resources and experience

	Resources Available						Professional Experience			
	Equipment		Space		Time		NHS Banding		Years exp	
Conf to perform LSR component	SMR	*P*-Value	SMR	*P*-Value	SMR	*P*-Value	SMR	*P*-Value	SMR	*P*-Value
Strength	0.095	0.341	0.092	0.356	0.098	329	**0.214** ^*^	**0.031**	0.022	0.825
NMC	0.074	0.458	0.094	0.346	**0.205** ^*^	**0.039**	**0.408** ^**^	**< 0.001**	**0.262** ^*^	**0.008**
MQA	0.124	0.216	**0.240** ^*^	**0.015**	**0.294** ^*^	**0.003**	**0.329** ^**^	**< 0.001**	**0.256** ^*^	**0.009**
Plyometrics	0.088	0.379	0.167	0.093	0.180	0.071	**0.238** ^*^	**0.016**	0.001	0.989
SSDs	− 0.057	0.568	− 0.071	0.479	− 0.195	0.060	− 0.060	0.551	− 0.080	0.426
PRE	**0.446** ^**^	**< 0.001**	**0.426** ^**^	**< 0.001**	**0.360** ^**^	**< 0.001**	**0.237** ^*^	**0.017**	0.107	0.285

*SMR* Spearman, Ro, *P* Probability, Figures in Bold: Statistically Significant, ^*^ Weak correlation (for figures where *P <* 0.05), ^**^Moderate correlation (for figures where *P <* 0.05), *Conf* Confidence, *LSR* Late-stage rehabilitation, *NHS* National Health Service, *NMC *Neuromuscular Contro, *MQA* Movement Quality Analysis, *SSDs* Sports Specific Drills, *PRE* Psychological Readiness Evaluation, *exp* Experience

## Discussion

### Summary of findings

To the authors knowledge, this is the first survey to evaluate NHS physiotherapists’ perspectives of the resources available, and their confidence, to deliver late-stage rehabilitation and RTS testing to ACL-R patients. Overall, respondents perceived there were insufficient resources available to deliver optimal late-stage ACL-R rehabilitation. Furthermore, only about one-half (53%) of respondents perceived that 60% or more of patients progressed to late-stage rehabilitation and RTS. Therefore, structural barriers (e.g. number of available appointments, pressure for discharge) may also limit patient access to late-stage ACL-R rehabilitation in the NHS.

Respondents reported using a wide range of poorly supported exercise-based and psychological readiness tools to inform RTS decision making. Despite this, most respondents were confident to perform the exercise-based components of late-stage ACL-R rehabilitation (range 68–92%) and RTS testing (75%). By contrast, only 39% of respondents were confident to perform psychological readiness evaluation, which was consistently moderately positively correlated with their perspectives of resource availability.

91% of respondents had ≥ 3 years of post-graduate clinical experience and 56% reported having a special interest in ACL-R rehabilitation. However, 85% of respondents spent less than one quarter of their time with ACL-R patients. Therefore, while NHS physiotherapists may be adapting their late-stage ACL-R rehabilitation strategies to the available facilities, they may lack knowledge and/or experience using contemporary evidence-based protocols in practice. Further targeted training in contemporary evidence-based protocols and improved access to appropriate resources may be required for NHS physiotherapists to deliver optimal late-stage ACL-R rehabilitation.

### Component delivery of late-stage ACL-R rehabilitation

There may be insufficient resources to deliver optimal late-stage ACL-R in NHS physiotherapy practice: approximately one-half to three quarters agreed they had sufficient resources to undertake strength (49–60%), neuromuscular control (63–71%), movement quality analysis (53–57%) and plyometrics (48–52%); less than half agreed they could complete sports specific drills (range 18%−38%) and RTS (26%−33%) and psychological readiness (20–46%) testing. The perceived difficulty delivering sports specific drills was perhaps unsurprising as such procedures (e.g. 505 agility test) [5] require extensive space to perform and a lack of resources has been highlighted significant barrier to physiotherapist delivered late-stage ACL-R rehabilitation in previous studies [7, 8]. However, only about one-half of respondents self-reported that most ACL-R patients progress to late-stage rehabilitation and RTS. Therefore, consistent with other areas of physiotherapy practice [36], there may also be structural barriers within the NHS (e.g. number of available appointments and pressure to discharge patients) that limit patient access to late-stage ACL-R rehabilitation.

In agreement with other studies [7–10, 37], respondents utilised a variety of exercise-based tests to inform their RTS decision making, many of which did not follow best practice. For example, respondents most often used horizontal hop tests, which are user friendly and require little resource to perform [14, 38, 39], to evaluate RTS plyometrics. However, respondents rarely used vertically orientated plyometric tests (e.g. Counter movement or drop jump), which may be more useful for detecting deficits in knee function [2, 18, 40] and retraining reactive strength in ACL-R patients [41]. Similarly, respondents’ most used RTS strength test was MMT (68%), which has demonstrated poor accuracy and limited ability to evaluate strength changes [42]. The IKD is considered the gold standard for ACL-R assessment as it can quantify strength [43, 44]. However, an IKD is expensive, bulky, requires extensive clinician training, and is time consuming to utilise compared with MMT. Therefore, it is perhaps unsurprising that only 23% of respondents utilised one during RTS testing and may reinforce the lack of access to such equipment in NHS physiotherapy practice. Despite the perceived lack of resources available and variety of testing procedures reported, most respondents were confident to deliver the exercise-based components of late-stage rehabilitation (range 68–92%) and RTS testing (76%). Respondents’ confidence to deliver the exercise-based components of late-stage ACL-R rehabilitation were more often positively correlated with their clinical experience (particularly NHS banding) than their perspectives of available resources. However, the correlations were generally weak, and their clinical relevance remains uncertain, meaning conclusions cannot be drawn.

Psychological readiness is a well-documented barrier to successful RTS [45–47] and guidelines promote its’ assessment during late-stage ACL-R rehabilitation [3, 4]. However, almost one-third (32%) of respondents did not evaluate late-stage ACL-R psychological readiness, and it was the sole component that less than half of respondents (39%) were confident to perform. Interestingly, respondents’ confidence to perform psychological readiness evaluation demonstrated consistent moderate positive correlations with their perspectives of available resources. Furthermore, most respondents agreed that using psychological scales (56%) or PROMs (70%), which are commonly used to collect psychological data, improved their confidence to make RTS decisions. Therefore, perceived resource limitations maybe more influential of physiotherapists’ confidence to evaluate psychological readiness than exercise-based testing during late-stage ACL-R rehabilitation, which is notable as psychological testing requires less equipment and space to deliver. 92% (92%) of respondents used at least one PROM during RTS testing, which is the highest percentage reported in physiotherapy practice (range 17–72%) [7–10, 20, 37, 48]. However, the PROMs that respondents’ used were generally not the most appropriate for late-stage ACL-R patients. For example, most respondents used the VAS scale (68%) to inform their RTS decision making. However, the VAS is unidimensional and typically measures pain levels, which may not be the primary barrier to RTS for ACL-R patients. Conversely, evidence based PROMs that assess elements of psychological readiness, such as the IKDC [16] and the ACL-RSI [49] were used much less frequently by respondents (IDKC: 26%; ACL-RSI: 37%).

Despite respondents’ year of post graduate experience, most spent less than one quarter of their time with ACL-R patients and only half perceived that most ACL-R patients progressed to late-stage rehabilitation in the NHS. Taken together, the findings suggest limited knowledge and/or experience in implementing late-stage ACL-R protocols in practice in addition to resource limitations. As such, NHS physiotherapists may benefit from further targeted training and opportunities to practice utilising evidence-based exercise based late-stage ACL-R rehabilitation protocols. Moreover, further in-depth exploration of physiotherapists knowledge gaps and their perceptions of the resources required to deliver optimal late-stage ACL-R rehabilitation is warranted.

### Strengths and limitations

This survey was conducted in line with recommended guidelines [30] which improved its transparency of reporting and internal validity, and its’ content was informed by contemporary evidence and received a good regional spread across the UK. However, The survey had several limitations. Firstly, there were less respondents than other international ACL-R physiotherapy surveys whose participant numbers ranged from 223 [50] −1074 [37] respectively. No data exists on how many NHS physiotherapists treat ACL-R patients so no power calculation could be performed. However, the study is likely underpowered. Secondly, although the survey’s content was informed by empirical research and the study teams’ knowledge and experiences of NHS practice, further tests (e.g. the Cutting Movement Assessment Score) or supplementary components of late-stage ACL-R rehabilitation (e.g. on field management) that are not routinely assessed in NHS departments were not captured. While this is somewhat mitigated by piloting the questions on NHS physiotherapists, the survey’s validity was not established. Thirdly, while the surveys narrow eligibility criteria provided in-depth insights into NHS physiotherapy practice, late-stage rehabilitation and RTS decision-making is inherently multidisciplinary. This focus is further compounded by recruiting via social media and professional networks which excluded NHS physiotherapists who don’t use e-platforms and meant the studies response rate could not be calculated. Therefore, a follow up study detailing the practices and perspectives of other stakeholders (e.g. patient or surgeon) that includes the components of late-stage rehabilitation that are not typically undertaken in the NHS (e.g. on field rehabilitation) may be beneficial. Finally, NHS physiotherapists with stronger views or a special interest in ACL-R maybe more likely to undertake the survey and the age and gender of respondents was not reported, which may have led the demographic analysis being underdeveloped.

## Conclusion

This survey identified the perspectives of resources available, and confidence, to deliver late-stage rehabilitation of NHS physiotherapists in ACL-R patients. Respondents perceived there were insufficient resources available to deliver optimal late-stage ACL-R rehabilitation in NHS physiotherapy practice and reported using a wide range of poorly supported exercise-based and psychological tools. However, most respondents were confident to deliver the exercise-based components of late-stage ACL-R rehabilitation (68–92%) and RTS testing (75%). In contrast, less than half (39%) of respondents were confident to perform late-stage psychological readiness evaluation. Targeted training in evidence-based protocols and improved access to appropriate facilities may be required for NHS physiotherapists to deliver optimal late-stage ACL-R rehabilitation.

## Supplementary Information

Additional file 1. CROSS checklist pdf.

Additional file 2. Survey Questions pdf.

Additional file 3. Survey responses pdf.

Additional file 4. Definitions of components of late-stage anterior cruciate ligament rehabilitation pdf.

## Data Availability

The datasets supporting the conclusions of this article are included within the article and its additional files.

## Abbreviations

ACL: Anterior Cruciate Ligament
ACL-R: Anterior Cruciate Ligament Reconstruction
ACL-RSI: Anterior Cruciate Ligament Return to Sports after Injury questionnaire
HHD: Hand-held dynamometry
IKD: Isokinetic dynamometry
IKDC: International Knee Documentation Committee
MMT: Manual muscle testing
MQA: Movement Quality Analysis
NHS: National Health Service
NMT: Neuromuscular Control
PRE: Psychological Readiness Evaluation
PROMs: Patient Reported Outcome Measures
RTS: Return to Sports
UK: United Kingdom
VAS: Visual analogue scale
