# Supplementary material for: Late-stage anterior cruciate ligament reconstruction rehabilitation in the United Kingdom: an online survey of National Health Service physiotherapists
Source: BMC Sports Sci Med Rehabil. 2025 Nov 22;18:46. doi: 10.1186/s13102-025-01438-2 (PMC12860011; doi:10.1186/s13102-025-01438-2)
Supplement: Supplementary file 2 — Additional file 2. Survey Questions pdf. [file 13102_2025_1438_MOESM2_ESM.docx]

**Additional File 2: Published Survey**

**Late stage rehabilitation & return to sports for ACL-R in the NHS: A Physiotherapy Survey**

**What is this survey about?**

Thank you for taking the time to consider completing this survey. 

It should take you **less than 10 minutes** to complete. 

We aim to explore NHS physiotherapists' perceptions of late stage rehabilitation and return to sports decision making. 

For the purpose of the survey please consider patients who have had:
Anterior Cruciate Ligament Reconstruction (ACL-R) surgery (with or without meniscal involvement), and are working towards returning to competitive sports. 

The survey aims to identify **NHS Physiotherapists** perceptions of:

- delivering late stage rehabilitation
- collecting return to sport outcome measures
- confidence and knowledge in this subject area

Participation in this survey is voluntary.  

It is important that, prior to participating in this survey, you have read the participant information sheet found below. Please click on the link and take time to read this information prior to proceeding. 

<https://1drv.ms/b/s!Ag7C63DYr1EVj2iD9fiR-DGdkxws> 

If you require any further information or have any further queries prior to completing the survey, you are welcome to contact the primary researcher, xxxxx

Section 1

ELECTRONIC CONSENT

By completing answers 1-5 you are agreeing

- You have read the participant information sheet
- You meet the inclusion criteria
- Are voluntarily agreeing to take part in this survey

1.I confirm I have read and understood the participant information sheet above and am happy to partipcate in the survey.

- Yes
- No

2.I confirm I am a Heath Care Professional Council (HCPC) registered physiotherapist practising in the United Kingdom?

- Yes
- No

3.I confirm within my role I treat more than 1 ACL reconstruction per year

- Yes
- No

4.I understand that I can submit **one** completed survey only.

- Yes
- No

Section 2

Physiotherapy profile

5.What is your current NHS job title? *If you work across more than one job please select the role in which you spend most of your time.*

- Band 5
- Band 6
- Band 7
- Band 8

6.How many years post-graduate experience do you have?

- Less than 3 years
- 3-6 years
- 7-10 years
- 11 -15 years
- 16 years-20 years
- Over 20 years

7.Would you describe yourself as having a special interest in ACL management?

- Yes
- No

8.What is your highest educational award?

- BSc
- Graduate Diploma Physiotherapy
- BA
- MSc (Pre-registration)
- Post Graduate Certificate
- Post Graduate Diploma
- MSc (Post-registration)
- MA
- MRes
- MPhil
- MBA
- PhD
- Professional Doctorate
- Other

9.Which region do you work in most of the time?

- Scotland
- North East
- North West
- Northern Ireland
- Yorkshire and Humber
- East Midlands
- West Midlands
- East of England
- Wales
- London
- South East
- South West

10.How much of your clinical time do you spend treating post op ACL-R?

- Less than 25% of the time
- Between 25-50% of the time
- Between 51- 75% of the time
- Over 76% of the time

Section 3

Incidence of returning to sports

For the purpose of this section please consider patients that return to **competitive**sports e.g. netball, rugby and football.

11.On average, **how many** of the ACL-R patients that you see progress onto late stage rehabilitation and subsequently, return to sports?

- 0-20%
- Between 21-40%
- Between 41-60%
- Between 61-80%
- Between 81-100%

12.On average **how long** after surgery do your ACL-R patients return to sports?

- 3-6 months
- 7-9 months
- 10-12 months
- 13months -2 years
- 2 years +

13.On average, **at which timeframe** might you commence late stage rehabilitation?

- 8 weeks post operatively
- 3 months post operatively
- 6 months post operatively
- 9 months post operatively
- 12 months post operatively

Section 4

Perceptions of NHS facilities for late stage ACL-R rehabilitation

This section will explore perceptions of NHS facilities used to provide late stage/ return to sports rehabilitation following ACL reconstruction surgery.  
Please state your level of agreement with the below statements.

14.The **equipment** in your NHS setting is adequate to provide these components of late stage rehabilitation

|  | Strongly disagree | Disagree | Neutral | Agree | Strongly agree |
| --- | --- | --- | --- | --- | --- |
| Strength |  |  |  |  |  |
| Neuromuscular control |  |  |  |  |  |
| Movement quality |  |  |  |  |  |
| Plyometric |  |  |  |  |  |
| Sports specific drills |  |  |  |  |  |
| Psychological readiness |  |  |  |  |  |

15.The **space** in your NHS setting is adequate to provide these components of late stage rehabilitation

|  | Strongly disagree | Disagree | Neutral | Agree | Strongly agree |
| --- | --- | --- | --- | --- | --- |
| Strength |  |  |  |  |  |
| Neuromuscular control |  |  |  |  |  |
| Movement quality |  |  |  |  |  |
| Plyometric |  |  |  |  |  |
| Sports specific drills |  |  |  |  |  |
| Psychological readiness |  |  |  |  |  |

16.The **time** in your NHS setting is adequate to provide these components of late stage rehabilitation

|  | Strongly disagree | Disagree | Neutral | Agree | Strongly agree |
| --- | --- | --- | --- | --- | --- |
| Strength |  |  |  |  |  |
| Neuromuscular control |  |  |  |  |  |
| Movement quality |  |  |  |  |  |
| Plyometric |  |  |  |  |  |
| Sports specific drills |  |  |  |  |  |
| Psychological readiness |  |  |  |  |  |

17.Do you have access to a **gym** to carry out late stage rehabilitation?

- Yes plus we offer an ACL rehab class
- Yes
- No

Section 5

Perceptions of confidence in late stage rehabilitation

This section to explore your confidence in prescribing late stage/ return to sports rehabilitation.

18.How confident do you feel in your **knowledge** of the following components of late stage rehabilitation?

|  | Very unconfident | Unconfident | Neutral | Confident | Very confident |
| --- | --- | --- | --- | --- | --- |
| Strength |  |  |  |  |  |
| Neuromuscular |  |  |  |  |  |
| Movement quality |  |  |  |  |  |
| Plyometric |  |  |  |  |  |
| Sports specific drills |  |  |  |  |  |
| Psychological readiness |  |  |  |  |  |

19.Using an **orthopaedic protocol** improves my confidence with the below components of late stage rehabilitation.

|  | Strongly agree | Agree | Neutral | Disagree | Strongly disagree |
| --- | --- | --- | --- | --- | --- |
| Strength |  |  |  |  |  |
| Neuromuscular |  |  |  |  |  |
| Movement quality |  |  |  |  |  |
| Plyometric |  |  |  |  |  |
| Sports specific drills |  |  |  |  |  |
| Psychological readiness |  |  |  |  |  |

Section 6

**Return to sports decision making**
P1: Patient reported outcome measures (PROM)

Physiotherapists may collect a series of outcome measures to help make a decision on a patient's readiness to return to sports. 
Please select any of the below patient reported outcome measures you use ACL-R return to sport decision making.

20.Do you use any of the below patient reported outcome measures?

|  | Yes | Sometimes | No |
| --- | --- | --- | --- |
| Knee Injury and Osteoarthritis Outcome Score (KOOS) |  |  |  |
| Lower Extremity Functional Scale (LEFS) |  |  |  |
| International Knee Documentation Committee (IKDC) |  |  |  |
| Lysholm scale |  |  |  |
| Short Form 36 (SF36) or Short Form 12 (SF12) |  |  |  |
| The Tegner activity scale |  |  |  |
| Cincinnati Knee Rating System (CKRS) |  |  |  |
| Single Assessment Numeric Evaluation (SANE) score |  |  |  |
| Visual Analogue Scale (VAS) |  |  |  |
| EuroQol-5D (EQ-5D) |  |  |  |
| ACL quality of life (ACL-QoL) |  |  |  |
| Marx activity scale |  |  |  |
| None |  |  |  |

21.Do you use any of these psychological readiness scales?

|  | Yes | Sometimes | No |
| --- | --- | --- | --- |
| ACL Return to Sports after Injury (ACL-RSI) |  |  |  |
| Athletic Coping Skills Inventory |  |  |  |
| Knee Self Efficacy Score (K-SES) |  |  |  |
| Tampa Scale of Kinesophobia (TSK-11) |  |  |  |
| NIH PROMIS |  |  |  |
| Psychovitality Scale |  |  |  |
| None |  |  |  |

22.Do PROM's improve physiotherapists confidence?
Please state your level of agreement with the below statements.

|  | Strongly agree | Agree | Neutral | Disagree | Strongly disagree |
| --- | --- | --- | --- | --- | --- |
| Using a patient reported outcome measure improves my confidence in return to sports decision making |  |  |  |  |  |
| Using a measure of psychological readiness improves my confidence in return to sports decision making |  |  |  |  |  |

Section 7

**Return to sports decision making**
**P2: Performance based tests**

This section will explore return to sports tests completed by NHS Physiotherapists. 
The research team acknowledges that there is some overlap between certain categories.

23.*Strength*
Do you use any of these tests to inform return to sports decision making?
Please select one or more answers

|  | Yes | Sometimes | No |
| --- | --- | --- | --- |
| Isokinetic testing E.g Cybex |  |  |  |
| Isometric testing E.g. Hand held dynamometer |  |  |  |
| Manual muscle testing |  |  |  |
| Leg press *(assessed in clinic)* |  |  |  |
| Leg press *(patient reported)* |  |  |  |
| Leg extension *(assessed in clinic)* |  |  |  |
| Leg extension *(patient reported)* |  |  |  |
| None |  |  |  |

24.*Neuromuscular*
Do you use any of these tests to inform your return to sports decision? 
Please select one or more answers

|  | Yes | Sometimes | No |
| --- | --- | --- | --- |
| Y balance |  |  |  |
| Star Excursion Balance Test (SEBT) |  |  |  |
| Functional Movement Screen (FMS) |  |  |  |
| Qualitative analysis of single leg loading (QASLS) |  |  |  |
| None |  |  |  |

25.*Movement quality analysis*
Do you use any of these tests to inform your return to sports decision? 
Please select one or more answers

|  | Yes | Sometimes | No |
| --- | --- | --- | --- |
| Tuck jump test |  |  |  |
| Qualitative Analysis of Single Leg Squat (QASLS) |  |  |  |
| My Jump 2 app |  |  |  |
| Jump Landing System |  |  |  |
| Landing Error Scoring System |  |  |  |
| None |  |  |  |

26.*Plyometric*
(Often terms functional tests) 
Do you use any of these tests to inform your return to sports decision? 
Please select one or more answers

|  | Yes | Sometimes | No |
| --- | --- | --- | --- |
| Tuck jump test |  |  |  |
| My Jump 2 app |  |  |  |
| Hop for distance |  |  |  |
| Cross over hop |  |  |  |
| Statement 5 |  |  |  |
| Triple hop |  |  |  |
| 6 metre timed hop |  |  |  |
| Medial hop for distance |  |  |  |
| Lateral hop for distance |  |  |  |
| Single leg counter movement jump (CMJ) |  |  |  |
| Drop jump |  |  |  |
| Side hop |  |  |  |
| Square hop |  |  |  |
| Force plates/ mats |  |  |  |
| Agility T test |  |  |  |
| Jump Landing System |  |  |  |
| None |  |  |  |

27.Do you use any other sports specific test to inform your return to sports decision? 
*If Yes please state in the text box provided*
*If No please type No*

|  |
| --- |

28.Consider you are completing the above return to sports testing
Do you agree or disagree with the below statements?

|  | Strongly agree | Agree | Neutral | Disagree | Strongly disagree |
| --- | --- | --- | --- | --- | --- |
| I have enough equipment to complete return to sport testing |  |  |  |  |  |
| I have enough space to complete return to sport testing |  |  |  |  |  |
| I have enough time to complete return to sports testing |  |  |  |  |  |

29.Considering all of the above.
How confident are you making the decision of when a patient is ready to return to sports?

- Extremely confident
- Somewhat confident
- Neutral
- Somewhat not confident
- Extremely not confident

Section 8

End of the survey

Thank you for considering to participate in this survey. Unfortunately due to either not meeting the inclusion criteria you will now exit the survey.

Section 9

Completion and end of survey

Thank you for taking the time to complete this survey.

As the data is anonymised at the point of submission, you will not be able to withdraw your responses once you submit your survey.

**If you have any queries or concerns about this survey, please contact xxxxxxxxx**
