# Supplementary material for: Late-stage anterior cruciate ligament reconstruction rehabilitation in the United Kingdom: an online survey of National Health Service physiotherapists
Source: BMC Sports Sci Med Rehabil. 2025 Nov 22;18:46. doi: 10.1186/s13102-025-01438-2 (PMC12860011; doi:10.1186/s13102-025-01438-2)
Supplement: Supplementary file 3 — Additional file 3. Survey responses pdf. [file 13102_2025_1438_MOESM3_ESM.docx]

## **Additional file 3: Survey Responses (from question 11)**

| Question | Responses | | | | | |
| --- | --- | --- | --- | --- | --- | --- |
| Q*.*11 On average, how many of the ACL-R patients that you see progress onto late stage rehabilitation and subsequently, return to sports?  *n=102* | 0-20% | Between 21-40% | | Between 41-60% | Between 61-80% | Between 81-100% |
|  | 11  (10.7%) | 10  (9.8%) | | 27  (26.4%) | 33  (32.3%) | 21  (20.5%) |
| Q*.*12 On average how long after surgery do your ACL-R patients return to sports?  *n=102* | 3-6 months | 7-9 months | | 10-12 months | 13 months- 2years | 2 years + |
|  | 2  (1.9%) | 15  (14.7%) | | 47  (46.1%) | 37  (36.2%) | 1  (0.9%) |
| Q*.*13 On average, at which timeframe might you commence late stage rehabilitation?  *n=102* | 8 weeks post operatively | 3 months post operatively | | 6 months post operatively | 9 months post operatively | 12 months post operatively |
|  | 3  (2.9%) | 11  (10.7%) | | 52  (50.9%) | 33  (32.4%) | 3  (2.9%) |
| Q*.*14 The equipment in your NHS setting is adequate to provide these components of late stage rehabilitation  *n=102* | Strongly agree | Agree | | Neutral | Disagree | Strongly disagree |
| Strength | 13  (12.7%) | 37  (36.3%) | | 7  (6.9%) | 25  (24.5%) | 20  (19.6%) |
| Neuromuscular control | 7  (6.9%) | 62  (60.8%) | | 15  (14.7%) | 13  (12.7%) | 5  (4.9%) |
| Movement quality | 8  (7.8%) | 49  (48.0%) | | 16  (15.7%) | 23  (22.5%) | 6  (5.9%) |
| Plyometric | 11  (10.8%) | 38  (37.3%) | | 21  (20.6%) | 26  (25.5%) | 6  (5.9%) |
| Sports specific drills | 4  (3.9%) | 16  (15.7%) | | 14  (13.7%) | 49  (48.0%) | 19  (18.6%) |
| Psychological readiness | 3  (2.9%) | 29  (28.4%) | | 30  (29.4%) | 32  (31.4%) | 8  (7.8%) |
| Q*.*15 The space in your NHS setting is adequate to provide these components of late stage rehabilitation  *n=102* | Strongly agree | Agree | | Neutral | Disagree | Strongly disagree |
| Strength | 13  (12.7%) | 48  (47.1%) | | 10  (9.8%) | 6  (15.7%) | 15  (14.7%) |
| Neuromuscular control | 13  (12.7%) | 59  (57.8%) | | 17  (16.7%) | 7  (6.9%) | 6  (5.9%) |
| Movement quality | 13  (12.7%) | 44  (43.1%) | | 20  (19.6%) | 17  (16.7%) | 8  (7.8%) |
| Plyometric | 14  (13.7%) | 39  (38.2%) | | 16  (15.7%) | 24  (23.5%) | 9  (8.8%) |
| Sports specific drills | 6  (5.9%) | 12  (11.8%) | | 11  (10.8%) | 37  (36.3%) | 36  (35.3%) |
| Psychological readiness | 10  (9.8%) | 37  (36.3%) | | 27  (26.5%) | 16  (15.7%) | 12  (11.8%) |
| Q*.*16 The time in your NHS setting is adequate to provide these components of late stage rehabilitation  *n=102* | Strongly agree | Agree | | Neutral | Disagree | Strongly disagree |
| Strength | 12  (11.8%) | 48  (47.1%) | | 11  (10.8%) | 22  (21.6%) | 9  (8.8%) |
| Neuromuscular control | 11  (10.8%) | 53  (52%) | | 11  (10.8%) | 19  (18.6%) | 8  (7.8%) |
| Movement quality | 11  (10.8%) | 43  (42.2%) | | 10  (9.8%) | 29  (28.4%) | 9  (8.8%) |
| Plyometric | 11  (10.8%) | 41  (40.2%) | | 14  (13.7%) | 27  (26.5%) | 9  (8.8%) |
| Sports specific drills | 8  (7.8%) | 31  (30.4%) | | 10  (9.8%) | 34  (33.3%) | 19  (18.6%) |
| Psychological readiness | 9  (8.8%) | 33  (32.4%) | | 19  (18.6%) | 31  (30.4%) | 10  (9.8%) |
| Q.17 Do you have access to a gym to carry out later stage rehabilitation?  *n=102* | Yes, plus we offer an ACL rehab class | Yes | | No |  |  |
|  | 53  (52.0%) | 33  (32.4%) | | 16  (15.7%) |  |  |
| Q.18 How confident do you feel in your knowledge of the following components of late stage rehabilitation?  *n=102* | Very confident | Confident | | Neutral | Unconfident | Very unconfident |
| Strength | 29  (28.4%) | 65  (63.7%) | | 4  (3.9%) | 3  (2.9%) | 1  (1.0%) |
| Neuromuscular control | 20  (19.6%) | 65  (63.7%) | | 11  (10.8%) | 4  (3.9%) | 1  (1.0%) |
| Movement quality | 22  (21.5%) | 58  (56.9%) | | 16  (15.7%) | 5  (4.9%) | 1  (1.0%) |
| Plyometric | 27  (26.5%) | 53  (51.9%) | | 17  (16.7%) | 4  (3.9%) | 1  (1.0%) |
| Sports specific drills | 17  (17.6%) | 52  (53.9%) | | 18  (17.6%) | 9  (8.8%) | 2  (2.0%) |
| Psychological readiness | 7  (6.9%) | 33  (32.4%) | | 33  (32.4%) | 24  (23.5%) | 5  (4.9%) |
| Q.19 Using an orthopaedic protocol improves my confidence with the below components of late stage rehabilitation.  *n=102* | Strongly agree | Agree | | Neutral | Disagree | Strongly disagree |
| Strength | 10  (9.8%) | 38  (37.3%) | | 23  (22.5%) | 23  (22.5%) | 8  (7.8%) |
| Neuromuscular control | 9  (8.8%) | 30  (29.4%) | | 27  (26.5%) | 29  (28.4%) | 7  (6.9%) |
| Movement quality | 7  (6.9%) | 30  (29.4%) | | 28  (27.5%) | 30  (29.4%) | 7  (6.9%) |
| Plyometric | 8  (7.8%) | 36  (35.3%) | | 16  (15.7%) | 35  (34.3%) | 7  (6.9%) |
| Sports specific drills | 5  (4.9%) | 26  (25.5%) | | 24  (23.5%) | 38  (37.3%) | 19  (18.8%) |
| Psychological readiness | 4  (3.9%) | 21  (20.6%) | | 26  (25.5%) | 35  (34.3%) | 16  (15.7%) |
| Q. 20 Do you use any of the below patient reported outcome measures (PROM)? | Yes | Sometimes | | No |  |  |
| Knee Injury and Osteoarthritis Outcome Score (KOOS)  *n=97* | 34  (35.1%) | 28  (28.9%) | | 35  (36.1%) |  |  |
| Lower Extremity Functional Scale (LEFS)  *n=93* | 23  (24.7%) | 18  (19.4%) | | 52  (55.9%) |  |  |
| International Knee Documentation Committee (IKDC)  *n=95* | 25  (26.3%) | 8  (8.4%) | | 62  (65.3%) |  |  |
| Lysholm scale  *n=92* | 10  (10.9%) | 7  (7.6%) | | 75  (81.5%) |  |  |
| Short Form 36 (SF36) or Short Form 12 (SF12)  *n=92* | 6  (6.5%) | 7  (7.6%) | | 79  (85.9%) |  |  |
| The Tegner activity scale  *n=92* | 8  (8.7%) | 6  (6.5%) | | 78  (84.8%) |  |  |
| Cincinnati Knee Rating System (CKRS)  *n=90* | 4  (4.4%) | 6  (6.7%) | | 80  (88.9%) |  |  |
| Single Assessment Numeric Evaluation (SANE) score  *n=91* | 2  (2.2%) | 5  (5.5%) | | 84  (92.3%) |  |  |
| Visual Analogue Scale (VAS)  *n=98* | 67  (68.4%) | 20  (20.4%) | | 11  (11.2%) |  |  |
| EuroQol-5D (EQ-5D)  *n=93* | 22  (23.7%) | 13  (14.0%) | | 58  (62.4%) |  |  |
| ACL quality of life (ACL-QoL)  *n=92* | 12  (13.0%) | 15  (16.3%) | | 65  (70.7%) |  |  |
| Marx activity scale  *n=91* | 0  (0.0%) | 3  (3.3%) | | 88  (96.7%) |  |  |
| None  *n=67* | 5  (7.5%) | 8  (11.9%) | | 54  (80.6%) |  |  |
| Q. 21 Do you use any of these psychological readiness scales? | Yes | Sometimes | | No |  |  |
| ACL Return to Sports after Injury (ACL-RSI)  *n=97* | 36  (37.1%) | 10  (10.3%) | | 51  (52.6%) |  |  |
| Athletic Coping Skills Inventory  *n=90* | 0  (0.0%) | 2  (2.2%) | | 88  (97.8%) |  |  |
| Knee Self Efficacy Score (K-SES)  *n=91* | 6  (6.6%) | 6  (6.6%) | | 79  (86.8%) |  |  |
| Tampa Scale of Kinesophobia (TSK-11)  *n=93* | 13  (14.0%) | 10  (10.8%) | | 70  (75.3%) |  |  |
| NIH PROMIS  *n=89* | 0  (0.0%) | 1  (1.1%) | | 88  (98.9%) |  |  |
| Psychovitality Scale  *n=90* | 0  (0.0%) | 1  (1.1%) | | 89  (98.9%) |  |  |
| None  *n=76* | 24  (31.6%) | 5  (6.6%) | | 47  (61.8%) |  |  |
| Q. 22 Do PROM's improve physiotherapists confidence? Please state your level of agreement with the below statements.  *n=102* | Strongly agree | Agree | | Neutral | Disagree | Strongly disagree |
| Using a patient reported outcome measure improves my confidence in return to sports decision making. | 17  (16.7%) | 55  (53.9%) | | 21  (20.6%) | 9  (8.8%) | 0  (0.0%) |
| Using a measure of psychological readiness improves my confidence in return to sports decision making | 21  (20.6%) | 36  (35.3%) | | 37  (36.3%) | 7  (6.9%) | 1  (1.0%) |
| Q.23  Strength Do you use any of these tests to inform return to sports decision making? Please select one or more answers | Yes | Sometimes | | No |  |  |
| Isokinetic testing E.g Cybex  *n=93* | 21  (22.6%) | 4  (4.3%) | | 68  (73.1%) |  |  |
| Isometric testing E.g. Hand held dynamometer  *n=95* | 22  (23.2%) | 15  (15.8%) | | 58  (61.1%) |  |  |
| Manual muscle testing  *n=100* | 68  (68.0%) | 14  (14.0%) | | 18  (18.0%) |  |  |
| Leg press (assessed in clinic)  *n=97* | 46  (47.4%) | 10  (10.3%) | | 41  (42.3%) |  |  |
| Leg press (patient reported)  *n=95* | 38  (40%) | 34  (35.8%) | | 23  (24.2%) |  |  |
| Leg extension (assessed in clinic)  *n=97* | 46  (47%) | 10  (12.5%) | | 41  (46.9%) |  |  |
| Leg extension (patient reported)  *n=96* | 44  (45.8%) | 27  (28.1%) | | 25  (26.0%) |  |  |
| None  *n=61* | 14  (23.0%) | 8  (13.1%) | | 39  (63.9%) |  |  |
| Q. 24  Neuromuscular  Do you use any of these tests to inform your return to sports decision?  Please select one or more answers | Yes | Sometimes | | No |  |  |
| Y balance  *n=92* | 36  (39.1%) | 20  (21.7%) | | 36  (39.1%) |  |  |
| Star Excursion Balance Test (SEBT)  *n=96* | 57  (59.4%) | 22  (22.9%) | | 17  (17.7%) |  |  |
| Functional Movement Screen (FMS)  *n=97* | 20  (20.6%) | 17  (17.5%) | | 60  (61.9%) |  |  |
| Qualitative analysis of single leg loading (QASLS)  *n=93* | 45  (48.4%) | 14  (15.1%) | | 34  (36.6%) |  |  |
| None  *n=69* | 4  (5.8%) | 3  (4.3%) | | 62  (89.9%) |  |  |
| Q. 25 Movement quality analysis  Do you use any of these tests to inform your return to sports decision?  Please select one or more answers | Yes | Sometimes | | No |  |  |
| Tuck jump test  *n=93* | 19  (20.4%) | 11  (11.8%) | | 63  (67.7%) |  |  |
| Qualitative Analysis of Single Leg Squat (QASLS)  *n=92* | 55  (59.8%) | 14  (15.2%) | | 23  (25.0%) |  |  |
| My Jump 2 app  *n=89* | 7  (7.9%) | 8  (9.0%) | | 74  (83.1%) |  |  |
| Jump Landing System  *n=90* | 12  (13.3%) | 5  (5.6%) | | 73  (81.1%) |  |  |
| Landing Error Scoring System  *n=91* | 8 (8.8%) | 13  (14.3%) | | 70  (76.9%) |  |  |
| None  *n=71* | 11  (15.5%) | 6  (8.5%) | | 54  (76.1%) |  |  |
| Q. 26 Plyometric  (Often terms functional tests)  Do you use any of these tests to inform your return to sports decision?  Please select one or more answers | Yes | Sometimes | | No |  |  |
| Tuck jump test  *n=91* | 16  17.6% | 11  (12.1%) | | 64  (70.3%) |  |  |
| My Jump 2 app  *n=91* | 5  (5.4%) | 8  (9.2%) | | 78  (85.7%) |  |  |
| Hop for distance  *n=99* | 78  (78.8%) | 10  (10.1%) | | 11  (11.1%) |  |  |
| Cross over hop  *n=100* | 62  (62.0%) | 12  (12.0%) | | 26  (26.0%) |  |  |
| Triple hop  *n=98* | 70  (71.4%) | 11  (11.2%) | | 17  (17.3%) |  |  |
| 6 metre timed hop  *n=94* | 32  (34.0%) | 10  (10.6%) | | 52  (55.3%) |  |  |
| Medial hop for distance  *n=91* | 25  (27.5%) | 10  (11.0%) | | 56  (61.5%) |  |  |
| Lateral hop for distance  *n=92* | 27  (29.3%) | 10  (10.9%) | | 55  (59.8%) |  |  |
| Single leg counter movement jump (CMJ)  *n=92* | 13  (14.1%) | 10  (10.9%) | | 69  (75.0%) |  |  |
| Drop jump  *n=91* | 30  (33.0%) | 20  (22.0%) | | 41  (45.1%) |  |  |
| Side hop  *n=93* | 30  (32.3%) | 19  (20.4%) | | 44  (47.3%) |  |  |
| Square hop  *n=90* | 14  (15.6%) | 17  (18.9%) | | 59  (65.6%) |  |  |
| Force plates/ mats  *n=90* | 3  (3.3%) | 2  (2.2%) | | 85  (94.4%) |  |  |
| Agility T test  *n=89* | 16  (18.0%) | 16  (18.0%) | | 57  (64.0%) |  |  |
| Jump Landing System  *n=89* | 3  (3.4%) | 2  (2.2%) | | 84  (94.4%) |  |  |
| None  *n=68* | 2  (2.9%) | 3  (4.4%) | | 63  (92.6%) |  |  |
| Q. 27 Do you use any other sports specific test to inform your return to sports decision?  If Yes please state in the text box provided  If No please type No | | | | | | |
| *No* | | | 74 (72.5%) | | | |
| *Sport dependent tasks* | | | 6 (5.8%) | | | |
| *Melbourne RTS* | | | 3 (2.9%) | | | |
| *All tests are completed in our ACL class* | | | 3 (2.9%) | | | |
| *30m line, lateral hop* | | | 1 (0.9%) | | | |
| *Double leg counter movement jump* | | | 1 (0.9%) | | | |
| *Sprint training protocols* | | | 1 (0.9%) | | | |
| *SAQ* | | | 1 (0.9%) | | | |
| *CMAS* | | | 1 (0.9%) | | | |
| *Single leg squat, bridge and calf raise* | | | 1 (0.9%) | | | |
| *Self reported confidence* | | | 1 (0.9%) | | | |
| *Edgern tests* | | | 1 (0.9%) | | | |
| *30 sec hop frontal plane, STARRT* | | | 1 (0.9%) | | | |
| *Bleep test* | | | 1 (0.9%) | | | |
| *I rarely see people at this stage* | | | 1 (0.9%) | | | |
| *Difficult to do any due to space and equipment* | | | 1 (0.9%) | | | |
| *505 agility drills* | | | 1 (0.9%) | | | |
| *Participation in sports* | | | 1 (0.9%) | | | |
| Q. 28 Consider you are completing the above return to sports testing Do you agree or disagree with the below statements?  *n=102* | Strongly agree | Agree | | Neutral | Disagree | Strongly disagree |
| I have enough equipment to complete return to sport testing | 4  (3.9%) | 22  (21.6%) | | 16  (15.7%) | 46  (45.1%) | 14  (13.7%) |
| I have enough space to complete return to sport testing | 8  (7.8%) | 20  (19.6%) | | 10  (9.8%) | 39  (38.2%) | 25  (24.5%) |
| I have enough time to complete return to sports testing | 6  (5.9%) | 28  (27.5%) | | 17  (16.7%) | 31  (30.4%) | 20  (19.6%) |
| Q. 29  Considering all of the above. How confident are you making the decision of when a patient is ready to return to sports?  *n=102* | Extremely confident | Somewhat confident | | Neutral | Somewhat not confident | Extremely not confident |
|  | 10  (9.8%) | 67  (65.7%) | | 12  (11.8%) | 11  (10.8%) | 2  (2.0%) |
