# Supplementary material for: Late-stage anterior cruciate ligament reconstruction rehabilitation in the United Kingdom: an online survey of National Health Service physiotherapists
Source: BMC Sports Sci Med Rehabil. 2025 Nov 22;18:46. doi: 10.1186/s13102-025-01438-2 (PMC12860011; doi:10.1186/s13102-025-01438-2)
Supplement: Supplementary file 4 — Additional file 4. Definitions of components of late-stage anterior cruciate ligament rehabilitation pdf. [file 13102_2025_1438_MOESM4_ESM.docx]

**Additional file 4: Definitions of components of late-stage anterior cruciate ligament rehabilitation**

| Component | Definition |
| --- | --- |
| **Strength** | Training programs involving concentric, eccentric, isometric and isotonic exercises (1).  Training likely utilises equipment such as leg press and leg extension machines, free weights and Therabands (2) |
| **Neuromuscular control** | The ability of the nervous system and muscles to work together to generate force and maintain dynamic stability in functioning joints (3, 4) and integrates essential movement patterns such as balance, core stability, dynamic control, and agility training (5, 6)  Neuromuscular training commonly utilises equipment such as foam mats, BOSU balls, gym balls, and wobble boards (2). |
| **Movement quality** | Functional movement patterns/kinematics which assess and/or address the quality and coordination of joint and body segment motion during activity e.g. dynamic knee valgus, trunk sway, and change of direction.  Rehabilitation usually targets these patterns using internal or external cues (7). |
| **Plyometrics** | Exercises involve a stretch-shortening cycle (i.e. a rapid lengthening of a muscle tendon unit immediately followed by a rapid shortening (8).  Plyometric training commonly includes hop tests and other strategies to retrain explosive performance in sports (9, 10). |
| **Sports specific drills** | Rehabilitation and testing on specific demands of that sport i.e. throwing/ tackling.  Sports specific drills aim to bridge a person’s transition back to the sporting environment |
| **Psyhcological Readiness**  **Evaluation** | Psychological factors that impact on the athlete’s ability to return to previous level of activity and include fear of re-injury/kinesiophobia and confidence/self-efficacy (6, 11, 12) |

Note: Several definitions taken verbatim from source

1. American College of Sports M. American College of Sports Medicine position stand. Progression models in resistance training for healthy adults. Med Sci Sports Exerc. 2009;41(3):687-708.

2. Dunphy E, Hamilton FL, Button K, Murray E. A scoping review of the resources needed to deliver anterior cruciate ligament physiotherapy rehabilitation in randomised controlled trials. Physical Therapy Reviews. 2020;25(2):81-95.

3. Buckthorpe M. Optimising the Late-Stage Rehabilitation and Return-to-Sport Training and Testing Process After ACL Reconstruction. Sports Med. 2019;49(7):1043-58.

4. Akbar S, Soh KG, Jazaily Mohd Nasiruddin N, Bashir M, Cao S, Soh KL. Effects of neuromuscular training on athletes physical fitness in sports: A systematic review. Front Physiol. 2022;13:939042.

5. Myer GD, Faigenbaum AD, Ford KR, Best TM, Bergeron MF, Hewett TE. When to initiate integrative neuromuscular training to reduce sports-related injuries and enhance health in youth? Curr Sports Med Rep. 2011;10(3):155-66.

6. Diermeier TA, Rothrauff BB, Engebretsen L, Lynch A, Svantesson E, Hamrin Senorski EA, et al. Treatment after ACL injury: Panther Symposium ACL Treatment Consensus Group. Br J Sports Med. 2021;55(1):14-22.

7. van Melick N, van Cingel RE, Brooijmans F, Neeter C, van Tienen T, Hullegie W, et al. Evidence-based clinical practice update: practice guidelines for anterior cruciate ligament rehabilitation based on a systematic review and multidisciplinary consensus. Br J Sports Med. 2016;50(24):1506-15.

8. Davies G, Riemann BL, Manske RC. Current Concepts of Plyometric Exercise. Int J Sports Phys Ther. 2015;10(6):760-86.

9. Ebert JR, Du Preez L, Furzer B, Edwards P, Joss B. Which Hop Tests Can Best Identify Functional Limb Asymmetry in Patients 9-12 Months After Anterior Cruciate Ligament Reconstruction Employing a Hamstrings Tendon Autograft? Int J Sports Phys Ther. 2021;16(2):393-403.

10. Grindem H, Snyder-Mackler L, Moksnes H, Engebretsen L, Risberg MA. Simple decision rules can reduce reinjury risk by 84% after ACL reconstruction: the Delaware-Oslo ACL cohort study. Br J Sports Med. 2016;50(13):804-8.

11. Moksnes H, Ardern CL, Kvist J, Engebretsen L, Risberg MA, Myklebust G, et al. Assessing implementation, limited efficacy, and acceptability of the BEAST tool: A rehabilitation and return-to-sport decision tool for nonprofessional athletes with anterior cruciate ligament reconstruction. Phys Ther Sport. 2021;52:147-54.

12. Association TBAfSotKaTBOSTaA. BOA, BASK, BOSTAA Elective Care Standards: Best Practice for Management of Anterior Cruciate Ligament (ACL) Injuries Available from: <https://wwwboaacuk/static/88a4c3e3-df3e-4e51-a92e7d2f86d7d82a/Best-Practice-Book-for-management-of-Anterior-Cruciate-Ligament-injuriespdf>. 2023.
